# Evaluating the Diagnostic Potential of Serum Vascular Endothelial Growth Factor and Adiponectin in Diabetic Peripheral Neuropathy

**DOI:** 10.7759/cureus.53017

**Published:** 2024-01-26

**Authors:** Palani Selvam Mohanraj, Arani Das, Aniruddha Sen, Amit Ranjan, Vinoth Rajendran, Anupriya Velu, U Venkatesh

**Affiliations:** 1 Biochemistry, All India Institute of Medical Sciences, Gorakhpur, Gorakhpur, IND; 2 Physiology, All India Institute of Medical Sciences, Gorakhpur, Gorakhpur, IND; 3 Physical Medicine and Rehabilitation, All India Institute of Medical Sciences, Gorakhpur, Gorakhpur, IND; 4 Community Medicine and Family Medicine, All India Institute of Medical Sciences, Gorakhpur, Gorakhpur, IND; 5 Biochemistry, Mahayogi Gorakhnath University Gorakhpur, Gorakhpur, IND

**Keywords:** vegf, biomarkers, diabetes neuropathy examination score, neuropathy symptom score, adiponectin, vascular endothelial growth factor, diabetic peripheral neuropathy

## Abstract

Introduction: Diabetic peripheral neuropathy (DPN) presents a formidable health challenge in type 2 diabetes mellitus (T2DM) patients. This study in eastern Uttar Pradesh aims to assess the roles of vascular endothelial growth factor (VEGF) and adiponectin in DPN, recognizing the crucial need for understanding its molecular underpinnings for enhanced diagnosis and management.

Methods: In a cross-sectional study analyzing clinical and biochemical data, 86 individuals aged 35 to 65 years were examined, including 43 with neuropathy and 43 without. Neuropathy assessment included the neuropathy symptom score (NSS), diabetes neuropathy examination (DNE) score, and nerve conduction studies. Levels of VEGF and adiponectin were correlated with motor nerve amplitude, NSS, and DNE scores. Receiver operating characteristic (ROC) curve analysis gauged diagnostic potential, and logistic regression assessed predictors for DPN.

Results: Patients with neuropathy exhibited significantly elevated VEGF levels compared to those without, while adiponectin showed no significant difference. VEGF demonstrated a negative correlation with motor nerve amplitude and a positive correlation with NSS and DNE scores. ROC analysis revealed strong diagnostic capability for VEGF (area under the curve: 0.807). NSS and DNE scores indicated good and moderate diagnostic accuracy, respectively. In logistic regression analysis, VEGF emerged as the sole significant predictor (odds ratio: 1.11, 95% CI (1.03, 1.20), p = 0.0092).

Conclusion: Findings suggest VEGF's potential as a biomarker for diagnosing DPN in T2DM, associated with neuropathy severity. Adiponectin showed no significant association. The study underscores NSS and DNE scores' therapeutic relevance as valid neuropathy assessment tools.

## Introduction

Diabetic peripheral neuropathy (DPN) emerges as a prevalent and devastating consequence of diabetes mellitus, affecting a considerable number of individuals globally. The epidemiological prevalence of DPN varies widely, affecting 6% to 51% of diabetic adults, with the variability linked to factors like age, disease duration, glycemic management, and whether the diabetes is type 1 or type 2. The impact of DPN on patients is significant, with clinical outcomes ranging from no symptoms to severe pain, and the potential for serious complications such as foot ulcers, which occur in 25% of these patients, and increased risk of amputation, underscoring the need for vigilant screening and proactive treatment [1]. The progressive loss of nerve fibers in both sensory and motor nerves characterizes DPN, leading to a spectrum of symptoms from pain and numbness to severe motor impairment [2]. The complicated pathophysiology of DPN encompasses metabolic and vascular changes, oxidative stress, and inflammation [3,4]. Despite the advances in understanding the pathophysiology of DPN, challenges remain in its early diagnosis and effective management. Current diagnostic methods, primarily based on clinical examination and nerve conduction studies, often identify the condition at an advanced stage, when interventions are less effective [5].

To strengthen our understanding and management of DPN, the identification of unique biomarkers has been a significant focus of research. Among these biomarkers, vascular endothelial growth factor (VEGF) and adiponectin have emerged as molecules of particular interest. VEGF, recognized for its key function in angiogenesis, has been linked to several diabetes outcomes, including neuropathy. Studies reveal that VEGF may contribute to neurovascular dysfunction in DPN [6,7]. Concurrently, adiponectin, an adipokine recognized for its anti-inflammatory and insulin-sensitizing capabilities, has been proposed to play a protective function in DPN. The possible neuroprotective properties of adiponectin and its ability to modulate inflammatory processes suggest fascinating possibilities for its participation in alleviating neuropathic consequences in diabetes [8].

Understanding the roles of VEGF and adiponectin is crucial for biomarker identification, early diagnosis, and targeted interventions. This study, conducted in eastern Uttar Pradesh, aims to provide insights into the significance of these biomarkers in DPN among individuals with type 2 diabetes mellitus (T2DM). The comprehensive neuropathy assessment includes the neuropathy symptom score (NSS) and diabetes neuropathy examination (DNE) score, along with nerve conduction studies. NSS evaluates symptomatology, encompassing pain, numbness, and other neuropathic symptoms, while the DNE score involves clinical examinations assessing reflexes, sensation, and muscle strength. Nerve conduction studies confirm neuropathy and gauge its severity.

Analyzing the associations of VEGF and adiponectin with disease severity and diagnostic value contributes to the development of more effective early detection and management strategies for DPN in diabetic populations. This approach integrates clinical, biochemical, and neuropathy assessments, providing a holistic understanding of the intricate interplay between these biomarkers and DPN pathogenesis in this specific population.

## Materials and methods

Study design and setting

This cross-sectional study was conducted in the Department of Biochemistry in collaboration with the Department of General Medicine at All India Institute of Medical Sciences, Gorakhpur, Gorakhpur, India. The study was approved by the Institutional Human Ethics Committee, All India Institute of Medical Sciences, Gorakhpur (IHEC ref no: IHEC/AIIMS-GKP/BMR/64/2022), and written informed consent was obtained from all individuals who participated in the study. Confidential management of participant data was assured, with data utilized exclusively for research purposes and safeguarded against unauthorized access. The research protocol adhered to the Indian Council of Medical Research Biomedical Guidelines for Human Participant Research, 2017.

Study population

This study involved 86 people divided into two groups: group 1 comprised 43 patients with T2DM and verified PNP, while group 2 consisted of 43 T2DM patients without neuropathy. The inclusion criteria for cases in the study consisted of patients aged 35 to 65 years with a diabetes duration of 1 to 20 years, recently diagnosed with PNP through clinical examination and confirmed by nerve conduction study. Controls were selected to match cases in terms of age, gender, and disease duration, with the exclusion criteria for both cases and controls including conditions such as stroke, cervical spondylosis, liver and kidney failure, infectious and inflammatory conditions, neoplasia, hypo/hyperthyroidism, use of antioxidant medication, pregnancy, lactation, and other neurological diseases. Cases specifically excluded patients with type 1 diabetes, while controls excluded patients with liver and kidney failure, infectious and inflammatory conditions, neoplasia, hypo/hyperthyroidism, antioxidant medication use, pregnancy, lactation, and other neurological diseases. The sample size of 43 participants per group was determined based on considerations of statistical power and confidence interval for a two-sample t-test. With an 80% power and a 95% confidence interval, the sample size was calculated to ensure sufficient precision in detecting significant differences between the groups. This calculation took into account the anticipated effect size, standard deviation, and the desired levels of significance and power.

Clinical and biochemical assessments

Demographic data, including age and gender, were gathered, and clinical and anthropometric parameters and the existence of co-morbidities will be recorded, according to a predesigned proforma. In this study, a standard blood sampling procedure was performed on the study subjects as a part of routine biochemical investigations after an overnight fast. A total of 6 ml of blood was collected from the antecubital vein following an overnight fast. The blood collection process involved using different vials for specific purposes: 2 ml of blood was placed in a fluoride vial to facilitate accurate glucose estimation, while another 2 ml of blood was collected in a plain vial for the separation of serum components. The serum was carefully separated from the blood cells and stored in cryovials at -20 degrees Celsius until it was ready for analysis. Additionally, 2 ml of blood was collected in an ethylenediaminetetraacetic acid vial, which served the purpose of measuring HbA1c levels. Routine biochemical parameters such as fasting blood glucose were assayed using the International Federation of Clinical Chemistry-recommended kit method using a fully automated clinical chemistry analyzer (Transasia XL 1000, Transasia Bio-Medicals Ltd., Mumbai, India). HbA1c will be assayed by HPLC using Biorad D10 Analyser (Biorad, Hercules, United States). Biochemical indicators, specifically fasting insulin (Monocent, Inc., United States), VEGF (Wuhan Fine Biotech Co., Ltd, Wuhan, China), and adiponectin levels (Diagnostics Biochem Canada Inc., London, Canada), were assessed using commercially available enzyme-linked immunosorbent assay kits as per the manufacturer's protocol. Homeostatic model assessment of insulin resistance was calculated from fasting glucose and insulin levels to serve as an indicator of insulin resistance.

Neuropathy assessment

Neuropathy evaluation included the modified NSS for symptom assessment, diabetes neuropathy examination (DNE) score for clinical examination and nerve conduction investigations to confirm neuropathy and quantify its severity. The modified NSS involved inquiring patients about sensations like numbness, abnormal temperature perceptions, tingling, burning pain, irritation from bedclothes on lower legs and feet, and nocturnal worsening of muscle cramps. Additional points were assigned for the presence of each symptom, with an extra point for the first five symptoms if nocturnal exacerbation occurred. The total score ranged up to 10 points, and a score exceeding 1 point was considered positive for PNP [9]. The diabetic neuropathy examination score (DNE) comprises eight items, including two for muscle strength, one for reflexes, and five for sensation. Each item is scored on a scale from 0 to 2, with 0 indicating normal function and 2 indicating severe disturbance. The total score can reach a maximum of 16 points. A positive diagnosis for PNP is defined when the score exceeds 3 points [10,11].

Statistical analysis

The data analysis was conducted using the IBM SPSS Statistics for Windows, Version 27 (Released 2020; IBM Corp., Armonk, New York, United States). Data normalization methods were done, and comparison analysis across groups applied suitable statistical tests. Correlation investigation explored the link between VEGF and adiponectin levels and neuropathy markers. Logistic regression analysis was incorporated to assess the predictive value of these biomarkers for neuropathy. Additionally, receiver operating characteristic (ROC) curve analysis analyzed the diagnostic capability of VEGF, adiponectin, NSS, and DNE scores for neuropathy, with the area under the curve (AUC) serving as an indicator of diagnostic efficiency.

## Results

The study encompassed 86 individuals categorized into two groups: group 1 with 43 patients diagnosed with T2DM and neuropathy, and group 2 with 43 T2DM patients without neuropathy. Demographic and clinical measurements, including age, gender, anthropometrics, blood pressure, and biochemical parameters, were assessed to characterize the study participants (Table 1).

**Table 1:** Clinical and biochemical characteristics of DM without neuropathy and DM with neuropathy Data are presented as mean ± SD or as numbers (%); * p < 0.05 HOMA-IR: Homeostatic model assessment of insulin resistance; VEGF: Vascular endothelial growth factor; NSS: Neuropathy symptom score; DNE: Diabetes neuropathy examination; CV: Conduction velocity; Amp: Amplitude; DM: Diabetes mellitus; BP: Blood pressure

Characteristic	DM with neuropathy (n = 43)	DM without neuropathy (n = 43)	p-value
Number of females	23 (53%)	19 (44%)	0.388
Age (years)	54.5±11	53.1±8.7	0.523
Fasting glucose (mg/dL)	166.1±56	152.5±40.7	0.2
Fasting insulin (µU/mL)	14.7±14.6	13.3±11.6	0.629
Weight (kg)	62.2±13.9	64.7±10.4	0.065
Height (cm)	154.6±10.5	156±9.6	0.529
Body mass index (kg/m²)	25.2±4.6	27.4±4.7	0.051
Waist (cm)	38.6±3.2	38.7±3.1	0.867
Hip (cm)	39.7±3.6	39.2±3.4	0.517
Waist hip ratio	0.99±0.03	0.98±0.04	0.854
Systolic BP (mmHg)	140.3±21.6	138.2±14.7	0.601
Diastolic BP (mmHg)	79.6±9.7	81.6±7	0.288
HbA1C (%)	7.8±1.8	7.2±1.2	0.052
HOMA-IR	6.6±7.6	4.8±4.3	0.176
VEGF (pg/mL)	35.1±12.6	27.9±9.4	0.004*
Adiponectin (µg/mL)	3.3±1.2	3.6±1.2	0.357
NSS	5.2±2.1	2.2±2.1	0.001*
DNE	1.5±0.8	0.9±0.8	0.001*
Motor CV (m/s)	51.7±12.4	67.1±35.4	0.010*
Sensory CV (m/s)	39.4±16.1	50.2±7.8	0.001*
Motor amp (mV)	7.4±2.6	11.5±3.2	0.001*
Sensory amp (mV)	20±11.9	24.7±11	0.065

Neuropathy was evaluated using the NSS and DNE scores, revealing higher scores in the neuropathy group, indicative of more severe symptoms and clinical signs. Nerve conduction studies confirmed neuropathy, demonstrating lower conduction velocities in patients with neuropathy.

VEGF exhibited significantly higher levels in patients with neuropathy, showing correlations with neuropathy severity indicators. Adiponectin levels showed no significant difference between groups and lacked correlations with neuropathy scores or nerve conduction parameters (Table 2).

**Table 2:** Correlation of VEGF and adiponectin with neuropathy symptom score and nerve conduction study * p < 0.05 VEGF: Vascular endothelial growth factor; NSS: Neuropathy symptom score; DNE: Diabetes neuropathy examination; CV: Conduction velocity; Amp: Amplitude

Parameter	VEGF		Adiponectin	
	Pearson correlation	p	Pearson correlation	p
NSS	0.238	0.028*	-0.068	0.536
DNE	0.071	0.518	0.074	0.498
Motor CV	0.037	0.732	-0.120	0.270
Sensory CV	-0.013	0.904	-0.079	0.470
Motor Amp	-0.301	0.005*	0.163	0.134
Sensory Amp	-0.120	0.271	-0.094	0.387

A logistic regression analysis was conducted to evaluate the likelihood of DPN based on several predictors including fasting glucose, fasting insulin, VEGF, adiponectin, HbA1C, and insulin resistance. The model was statistically significant and it explained 18.0% of the variance in neuropathy status and correctly classified 81.0% of cases. Sensitivity was 75.0%, specificity was 85.7%, positive predictive value was 82.0%, and negative predictive value was 80.0%. Of the predictors, only VEGF was statistically significant, with an odds ratio of 1.11 (95% CI (1.03, 1.20), p = 0.0092). Higher levels of VEGF were associated with an increased likelihood of having DPN. HbA1C approached significance, indicating a potential trend, with an odds ratio of 1.54 (95% CI (0.97, 2.46), p = 0.0656). Fasting glucose, fasting insulin, adiponectin, and insulin resistance were insignificant predictors in the model (Table 3).

**Table 3:** Logistic regression analysis predicting the likelihood of diabetic peripheral neuropathy * p < 0.05 B: Unstandardized regression coefficient; SE: Standard error; CI: Confidence interval; HOMA-IR: Homeostatic model assessment of insulin resistance; VEGF: Vascular endothelial growth factor

Predictor	B (SE)	Wald χ²	p	Odds ratio (95% CI)
Constant	-3.8106 (2.4033)	1.59	0.113	
Fasting glucose	-0.0123 (0.0090)	1.36	0.173	0.99 (0.97, 1.01)
Fasting insulin	-0.0852 (0.0753)	1.13	0.257	0.92 (0.77, 1.09)
VEGF	0.1049 (0.0403)	6.77	0.009*	1.11 (1.03, 1.20)
Adiponectin	-0.2495 (0.2188)	1.30	0.254	0.78 (0.51, 1.20)
HbA1C	0.4337 (0.2356)	3.39	0.066	1.54 (0.97, 2.46)
HOMA-IR	0.2492 (0.1821)	1.87	0.171	1.28 (0.90, 1.82)

**Figure 1:**
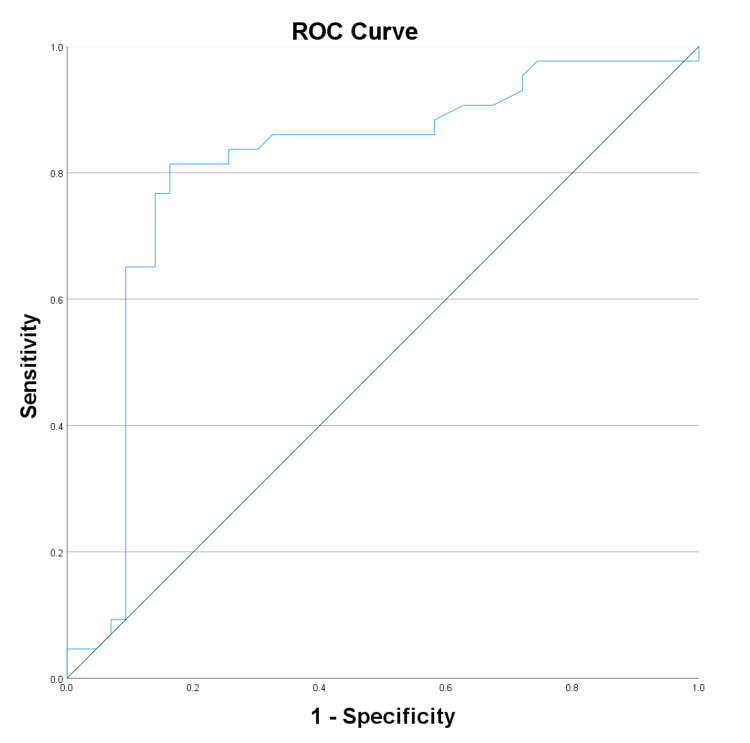
Receiver operating characteristic curve analysis for vascular endothelial growth factor ROC: Receiver operating characteristic

**Figure 2:**
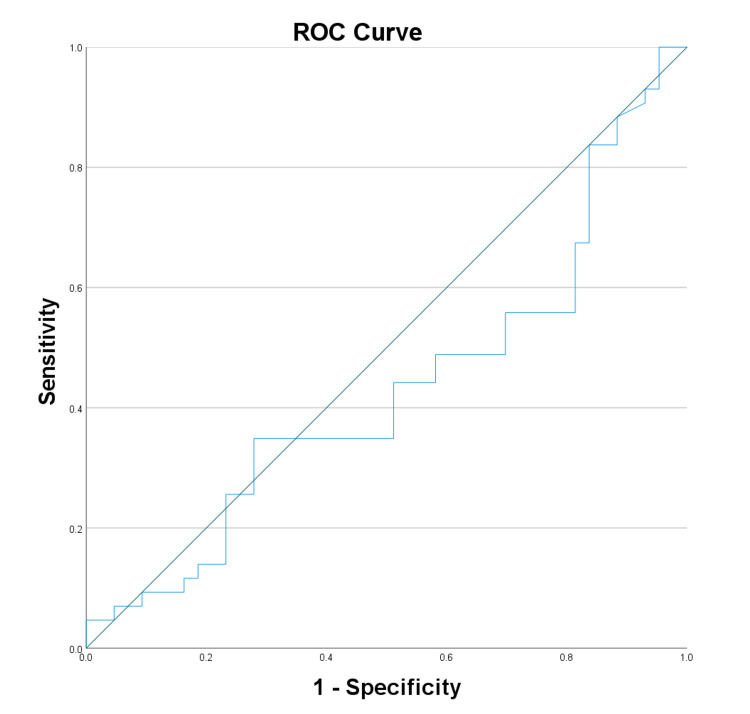
Receiver operating characteristic curve analysis for adiponectin ROC: Receiver operating characteristic

**Figure 3:**
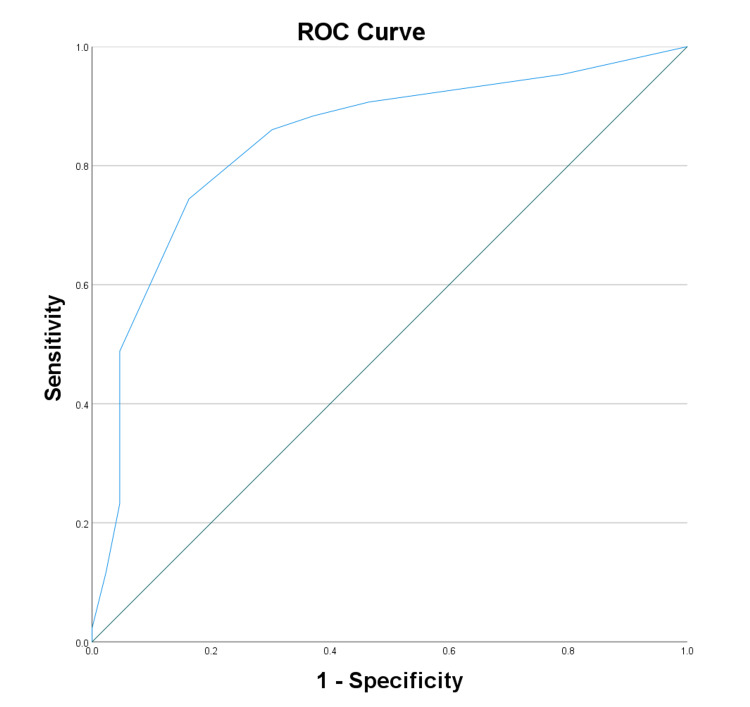
Receiver operating characteristic curve analysis for neuropathy symptom score ROC: Receiver operating characteristic

**Figure 4:**
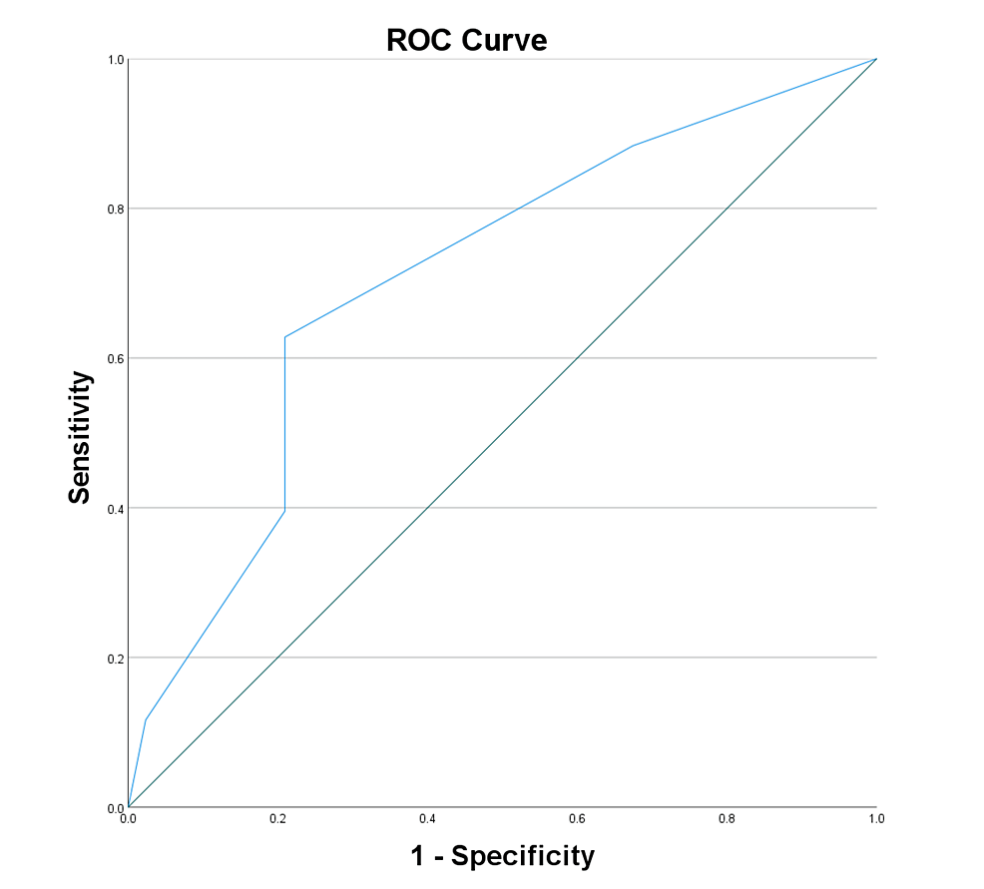
Receiver operating characteristic curve analysis for diabetic neuropathy examination score ROC: Receiver operating characteristic

The diagnostic potential of biomarkers and clinical scores for neuropathy was assessed by ROC curve analysis. VEGF displayed good diagnostic potential with an AUC of 0.807 (Figure 1), while adiponectin had a lower AUC of 0.431 (Figure 2). NSS showed high diagnostic accuracy (AUC: 0.841) (Figure 3), and the DNE score demonstrated moderate diagnostic utility (AUC: 0.707) (Figure 4, Table 4).

**Table 4:** ROC curve analysis of test variable: area under the curve * p < 0.05 a: Under the non-parametric assumption; b: Null hypothesis: true area = 0.5; VEGF: Vascular endothelial growth factor; NSS: Neuropathy symptom score; DNE: Diabetes neuropathy examination; ROC: Receiver operating characteristic

Test result variable(s)	Area	Standard error a	Asymptotic significant b	Asymptotic 95% confidence interval	
				Lower bound	Upper bound
VEGF	0.807	0.052	0.000*	0.706	0.908
Adiponectin	0.431	0.063	0.271	0.308	0.554
NSS	0.841	0.045	0.000*	0.753	0.929
DNE	0.707	0.057	0.001*	0.596	0.818

## Discussion

The study's important finding of higher VEGF levels in individuals with DPN gives a crucial insight into the pathophysiology of DPN in T2DM. This rise, plus the substantial associations of VEGF with neuropathy severity scores (NSS and DNE) and nerve conduction measures, accords with the concept that VEGF plays a multifunctional role in DPN.

VEGF is known for its angiogenic capabilities and its role in neurovascular coupling, suggesting that its dysregulation could contribute to the vascular and neurological dysfunctions characteristic of DPN [12]. The connection of VEGF with neuropathy severity highlights its potential as a biomarker for both the existence and progression of DPN. This is a noteworthy development in understanding DPN since it turns the focus towards a molecule that could be both a marker and a mediator of disease, creating opportunities for targeted therapeutics that could affect the disease course by influencing VEGF pathways. The findings have important clinical implications, especially considering the identification of VEGF as a possible biomarker. This discovery holds the capacity to transform early identification and intervention approaches for illnesses such as DPN. By employing VEGF as a biomarker, healthcare practitioners can augment their capacity to detect DPN at a premature phase, before the manifestation of symptoms. Early identification of DPN can result in prompt therapies that have the potential to prevent or alleviate the advancement of the condition and its related problems, such as the likelihood of developing foot ulcers and requiring amputations. Furthermore, the integration of VEGF as a diagnostic instrument could enable more individualized and focused treatment strategies, hence enhancing the overall control and quality of life for those suffering from diabetes. However, it is vital to interpret these data with an appreciation of the complicated biology of VEGF, which can have both protective and harmful effects in different settings. The challenge for future research is in identifying these roles more clearly and understanding how therapies targeting VEGF might play out in the clinical setting of DPN.

In contrast to VEGF, adiponectin did not reveal significant variations between the neuropathic and non-neuropathic groups, nor did it correlate with neuropathy severity. The literature on adiponectin's role in DPN reveals varying effects, indicating a complex relationship. Ji et al. suggest that adiponectin serves as a protective factor in preventing diabetes progression by suppressing inflammatory responses and increasing insulin sensitivity. This study also reported decreased adiponectin plasma levels in T2DM patients with DPN [13]. Furthermore, the neuroprotective effects of adiponectin have been proposed in various studies, indicating its potential role in alleviating neuropathic consequences in diabetes [14,15]. In contrast, Sun et al. in Chinese type 2 diabetes patients found a positive association between serum adiponectin levels and the presence of DPN [16]. The study acknowledges the controversial nature of the relationship between adiponectin and DPN in the existing literature. Notably, some studies found higher adiponectin levels in DPN patients [17], while others observed low adiponectin levels associated with DPN [13,18]. The mechanism by which serum adiponectin affects DPN remains unclear due to inconsistencies in reported outcomes.

The present study's divergence from some previous research conclusions could be influenced by factors such as sample size, demographics, and diagnostic criteria for DPN. Additionally, differences in ethnic compositions might impact adiponectin concentration levels, contributing to disparate findings. The multifaceted physiological functions of adiponectin, including its anti-inflammatory properties and potential impact on insulin sensitivity, make its role in DPN complex and warrant further investigation. However, the contrasting outcomes in the literature underscore the need for more extensive research, considering diverse populations and refining diagnostic criteria for DPN to elucidate the intricate relationship between adiponectin and diabetic neuropathy.

The study reinforces the value of the NSS and DNE scores in clinical settings, especially in resource-limited countries where advanced diagnostic tools are not readily available. The remarkable diagnostic accuracy of these scores in the study population highlights their usefulness in the early detection and monitoring of DPN. For physicians, our findings underscore the significance of comprehensive clinical examination in managing diabetic patients, especially considering the gradual development and progression of DPN.

Looking ahead, the study establishes a basis for future longitudinal research to track changes in biomarkers like VEGF over time in relation to the development and progression of DPN. It also throws up concerns regarding the molecular mechanisms underpinning VEGF’s participation in DPN, which could be vital for creating targeted therapeutics. Furthermore, investigations involving larger and more diverse populations are needed to corroborate these findings and explore potential ethnic or regional variations in DPN pathophysiology and biomarker profiles.

While the study provides useful insights, its cross-sectional approach limits the capacity to establish causal correlations. The geographical limitation to eastern Uttar Pradesh further limits the generalizability of the findings to other communities. Moreover, the sample size, although adequate for preliminary conclusions, should be enlarged in future studies to enhance the robustness of the results. To address these limitations and improve upon the current findings, future research should investigate doing longitudinal studies that allow for a more dynamic knowledge of VEGF and adiponectin levels across time. Expanding the study to encompass larger and more diverse populations will contribute to the generalizability of the results and strengthen the study's external validity. Additionally, researching the impact of additional possible biomarkers alongside VEGF and adiponectin could provide a more comprehensive knowledge of the complicated mechanisms involved in DPN.

## Conclusions

This study carries significance on numerous fronts. First, the finding of VEGF as a possible biomarker for DPN presents a prospective path for early detection and intervention. Elevated VEGF levels could serve as an early signal, enabling appropriate therapeutic measures. Second, the clinical evaluation tools, NSS and DNE scores, emerge as essential resources in ordinary clinical practice, particularly in countries with limited access to modern diagnostics. Third, the study contributes to the continuing discourse on adiponectin's involvement in DPN, challenging previous conceptions and demanding a reevaluation of its significance in different metabolic situations. Last, the findings underline the necessity for a holistic approach to understanding and controlling DPN, given the multidimensional interplay of numerous components.

In conclusion, this study considerably increases our understanding of DPN by emphasizing the potential of VEGF as a biomarker and showing the multifaceted role of adiponectin. The clinical implications underline the necessity of accessible diagnostic techniques, and the study lays the platform for future research to delve deeper into the molecular subtleties of DPN pathogenesis.
